# Combination therapy with PEG-IFN-*α* and 5-FU inhibits HepG2 tumour cell growth in nude mice by apoptosis of p53

**DOI:** 10.1038/sj.bjc.6604058

**Published:** 2007-10-30

**Authors:** S Hagiwara, M Kudo, T Nakatani, Y Sakaguchi, M Nagashima, N Fukuta, M Kimura, S Hayakawa, H Munakata

**Affiliations:** 1 Department of Gastroenterology and Hepatology, Kinki University School of Medicine, Osaka-Sayama, Japan; 2 Department of Pathology, Kinki University School of Medicine, Osaka-Sayama, Japan; 3 Department of Biochemistry, Kinki University School of Medicine, Osaka-Sayama, Japan

**Keywords:** hepatocellular carcinoma, pegylated interferon, 5-fluorouracil, p53, apoptosis

## Abstract

When the tumour suppressor p53 is activated by DNA damage, it stimulates the transcription of its target genes, which then induce cell cycle arrest or apoptosis. Here, we examined the role p53 plays in the antitumour effect of combination treatment with pegylated interferon (PEG-IFN)-*α* and 5-fluorouracil (5-FU), which has been shown to effectively treat advanced hepatocellular carcinoma (HCC). Nude mice were injected subcutaneously with cultured HepG2 cells, in which p53 is functional. They were treated a week later with PEG-IFN and/or 5-FU for 7 weeks, after which we measured and examined their tumours. Combination groups showed significantly lower tumour volumes and higher tumour cell apoptosis than the other groups. Combination treatment and PEG-IFN monotherapy also significantly elevated the p53 protein and mRNA levels in the tumour but only combination treatment increased the degree of p53 phosphorylation at serine46 and induced p53-regulated apoptosis-inducing protein 1 (*p53AIP1*) expression. The antitumour effects of combination treatment is due in part to the elevation by PEG-IFN of p53 protein and mRNA expression and in part to the DNA damage that is generated by 5-FU, which induces p53 serine46 phosphorylation, which in turn upregulates *p53AIP1* expression.

Hepatocellular carcinoma (HCC) has a poor prognosis worldwide. Although the treatment of choice is generally resection, patients with HCC that is unresectable due to advanced disease or hepatic dysfunction are treated by radiofrequency ablation (RFA) and arterial embolisation. However, none of these local treatments are suitable for patients whose HCC has invaded the portal vein. Recent studies have suggested that these patients may be effectively treated by combination therapy involving subcutaneous (s.c.) administration of interferon (IFN)-*α* and intrahepatic administration of 5-fluorouracil (5-FU) (Sakon *et al*, 2002; Obi *et al*, 2006). A Phase II trial has also revealed that continuous 5-FU infusion combined with thrice-weekly treatment with IFN-*α* effectively treats HCC, perhaps because the drugs together play a neoadjuvant role (Patt *et al*, 2003). Comprehensive genetic analyses with PCR arrays have revealed that this method may be useful for predicting whether combination chemotherapy with IFN-*α* and 5-FU can successfully treat HCC (Kurokawa *et al*, 2004). However, despite these observations, the molecular mechanisms behind the anti-HCC activity of IFN-*α*/5-FU combination therapy remain poorly understood.

The IFN-*α* group of closely related cytokines are typically produced early after infection with viruses and have antiviral and immunoregulatory activities (Samuel, 2001; Takaoka *et al*, 2003). They also have potent antitumour activities (Samuel, 2001; Takaoka *et al*, 2003). While studies examining the IFN-*α*-mediated signal transduction pathways have already identified a number of IFN-*α*-induced genes, it remains unclear whether these genes contribute to the antitumour activities of IFN-*α*. The IFN-*α* cytokines appear to exert their antitumour activities both indirectly by activating immune cells such as natural killer cells, macrophages, and dendritic cells (Biron, 2001; Belardelli *et al*, 2002), and directly by inducing apoptosis (Clemens, 2003). With regard to the latter activity, the consensus IFN-*α*, which is a non-naturally occurring type I IFN with higher specific activity than the natural type I IFNs, has been shown to suppress the growth of HCC both *in vitro* and *in vivo* (Hisaka *et al*, 2004). Additional studies have suggested that pegylated interferon (PEG-IFN)-*α*, where IFN-*α* is joined by an amide linkage to a 40 kDa branched polyethylene glycol (PEG), may have even better antitumour effects *in vivo*. For example, Bailon *et al* (2001) showed that PEG-IFN-*α* has more potent antitumour activity on human kidney-derived cancer cells *in vivo* than IFN-*α*. The pegylation of IFN appears to stably maintain an effective concentration of IFN in the blood (Perry *et al*, 2001).

The tumour suppressor gene *p53* is activated by the DNA damage that is induced by X-rays, ultraviolet (UV) rays, or anticancer drugs like 5-FU. Its protein then stimulates the transcription of its target genes, which induce cell cycle arrest or apoptosis. As a result, *p53* is frequently inactivated by mutations in tumour cells (Prives *et al*, 1999; Vogelstein *et al*, 2000; Vousden *et al*, 2002). Upon its phosphorylation at serine46, p53 also regulates the transcriptional activation of p53-regulated apoptosis-inducing protein 1 (p53AIP1), which probably plays an important role in mediating p53-dependent apoptosis (Oda *et al*, 2000). Recently, the combination of 5-FU and IFN-*α* was found to suppress HCC proliferation by elevating S-phase arrest and apoptosis (Kojiro *et al*, 2006). Given the central role p53 plays in the induction of cell cycle arrest and apoptosis, it is possible that the IFN-*α*/5-FU combination therapy activates p53 in some way. Supporting this is that IFN-*α* has been found to induce *p53* gene expression (Takaoka *et al*, 2003; Vilcek, 2003). To test whether p53 participates in the anti-HCC activity of IFN-*α*/5-FU combination therapy, we here treated nude mice carrying HepG2 cell tumours with 5-FU and/or IFN-*α*. HepG2 cells express functional p53. Our observations suggest that PEG-IFN-*α* elevates p53 protein expression, and that this, in combination with the DNA damage elicited by 5-FU, leads to enhanced HCC cell apoptosis *in vivo*.

## MATERIALS AND METHODS

### Cell lines and cell culture

The human HCC cell line HepG2 was obtained from the Health Science Research Resources Bank (Sennan, Japan) and was grown in Dullbecco's modified Eagle's medium (DMEM) (Sigma-Aldrich, St Louis, MO, USA) supplemented with 10% foetal bovine serum (FBS) (JRH Bioscience, St Lenexa, KS, USA) at 37°C under 5% CO_2_/95% air.

### Animals

Male BALB/c nude mice, aged 4 weeks, were purchased from Clea Japan, Inc. (Tokyo, Japan) and acclimated for a week.

### Compounds and study design

5-FU and PEG-IFN-*α* 2a (PEGASYS®) were supplied by Kyowa Hakko Kogyo Co., Ltd (Tokyo, Japan) and Chugai Pharmaceutical Co., Ltd. (Tokyo, Japan), respectively. The 5-week-old male BALB/c nude mice were injected s.c. with cultured HepG2 cells (10^6^ cells per mouse). When the tumour was 5–10 mm in diameter (7 days later), the mice were randomly divided into five groups of five: Group 1 (control group) received phosphate-buffered saline (PBS), Group 2 (5-FU group) received 10 mg kg^−1^ day^−1^ 5-FU, Group 3 (high-dose 5-FU) received 20 mg kg^−1^ day^−1^ 5-FU, Group 4 (PEG-IFN) received 1.5 mg kg^−1^ PEG-IFN-*α*, while Group 5 (combination) received 10 mg kg^−1^ 5-FU and 1.5 mg kg^−1^ PEG-IFN-*α*. Note that Groups 2, 4, and 5 can be directly compared because they received the same doses of the two compounds. 5-FU was delivered intraperitoneally (i.p.) five times a week while PEG-IFN-*α* was injected s.c. once a week. The animals were treated for 7 weeks. Tumour size was measured once a week in two directions by using calipers, and tumour volume was estimated by using the equation: length × (the width)^2^ (each week)/the length × (the width)^2^ (0 week). At the end of the experiments, the mice were killed under ether anaesthesia and their tumours were examined as detailed below. All animal procedures were performed according to approved protocols and in accordance with the recommendations for the proper care and use of laboratory animals. The Medical Ethics Committee of Kinki University School of Medicine approved the study (October 2004).

### Histopathological examination and detection of apoptosis

The tumours were resected and fixed in formalin. The tumour sections with the largest diameter were prepared as paraffin sections for haematoxylin and eosin (HE) staining. To detect apoptotic cells, the *In situ* Cell Death Detection Kit, TMR red (Roche Diagnostics GmbH, Mannheim, Germany) was used (TUNEL technology). The numbers of apoptotic cells in ten 1.35-mm^2^ areas of each HE-stained specimen where apoptotic cells were present at a relatively uniform density were determined under a fluorescence microscope. These counts were averaged to obtain the number of apoptotic cells per specimen.

### Immunoprecipitation and immunoblot analysis

Cell lysis, immunoprecipitation and immunoblotting were performed as described (Lehtonen *et al*, 2004). The p53 proteins were immunoprecipitated with an anti-p53 antibody (Ab-1; Oncogene Research Products, Cambridge, MA, USA) and immunoblotted by using rabbit IgG TrueBlot (eBioscience, San Diego, CA, USA) and anti-p53 or anti-p53-phospho-serine46 (Cell Signaling Technology, Danvers, MA, USA) antibodies. As a control, the supernatants obtained after p53 immunoprecipitation were directly subjected to immunoblotting with anti-*β*-actin antibody (Clone AC-74; Sigma-Aldrich, St Louis, MO, USA).

### RNA extraction and real-time RT–PCR

Total RNA was isolated from the HepG2 tumours by employing an RNeasy Mini kit (Qiagen, Hilden, Germany). Total RNA (200 *μ*g) was converted into cDNA in accordance with the manufacturer's instructions (ReverTra Ace, TOYOBO, Osaka, Japan). The reaction was performed at 30°C for 10 min, 42°C for 20 min, and 99°C for 5 min. Quantitative PCR was performed in 96-well plates by using the TaqMan probe assay (ABI Prism 7700; Perkin-Elmer, Waltham, MA, USA) (Eickhoff *et al*, 2000; Gautschi *et al*, 2001). For this, pairs of primers and TaqMan probes were designed by a Perkin-Elmer to amplify specific small fragments from p53 (Assay ID; Hs00153349) and p53AIP1 (Assay ID; Hs00223141). In addition, a pair of primers and a TaqMan probe designed for human glyceraldehyde-3-phosphate dehydrogenase (GAPDH) (Perkin-Elmer) were used as an internal standard of mRNA integrity within the experiment. The final reaction mixture contained 1 × TaqMan Universal PCR Master Mix (AmpliTaq Gold, AmpErase UNG, dNTP and dUTG, and optimised buffer) (Perkin-Elmer), 1 × p53 Mix or 1 × p53AIP1 Mix, and 1 × Control (GAPDH) Mix. The cycling conditions comprised an initial phase of 50°C for 2 and 10 min at 95°C that was followed by 50 cycles of 15 s at 95°C and 1 min at 60°C.

### Statistical analyses

The data are expressed as means±standard deviation. The statistical significance of differences between two groups was determined by Student's *t*-test. A probability value of 0.05 or less was considered to be significant.

## RESULTS

### HepG2 tumour cell growth in nude mice

The Group 3 mice, which received high-dose 5-FU (20 mg kg^−1^ day^−1^), were excluded from the following analyses because they all died within 3 weeks of treatment.

**Figure 1:**
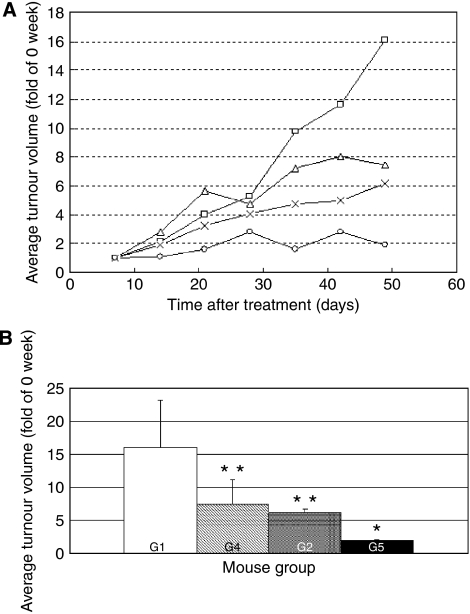
Tumour volumes of 5-FU- and/or PEG-IFN-*α*-treated nude mice bearing s.c. HepG2 cell tumours. Mice bearing 5–10 mm diameter s.c. HepG2 cell tumours were treated with PBS (G1, control group), 5-FU at 10 (G2, 5-FU) or 20 (G3, high-dose 5-FU) mg kg^−1^ day^−1^ only, PEG-IFN-*α* at 1.5 (G4, PEG-IFN), or with both at 10 mg kg^−1^ day^−1^ 5-FU and 1.5 mg kg^−1^ week^−1^ PEG-IFN-*α* (G5, combination). (**A**) Change in tumour volume over time. G1 (□), G2 (×), G4 (Δ), and G5 (○). The G3 group is not shown because these mice did not survive beyond 3 weeks of treatment. (**B**) Tumour volumes at the end of the experiment after 7 weeks of treatment. ^*^Statistically significant difference compared with G1, G2, and G4 (*P*<0.05). ^**^Statistically significant difference compared with G1 (*P*<0.05).

High-dose 5-FU also induced marked weight loss (date not shown). In contrast, treatment with 10 mg kg^−1^ day^−1^ 5-FU and/or 1.5 mg kg^−1^week^−1^ PEG-IFN-*α* did not affect the body weight of the mice (data not shown). The volumes of the tumours over the 7 weeks of treatment are shown in Figure 1A. When we compared the tumour volumes at the end of the experiment, Group 2 (5-FU), Group 4 (PEG-IFN), and Group 5 (combination) showed significantly lower tumour volumes than the control (Group 1). Furthermore, the tumour volumes of the combination group were significantly lower than those of the 5-FU and PEG-IFN monotherapy groups (*P*<0.05, Figure 1B).

### Histopathological examination and detection of apoptosis

**Figure 2:**
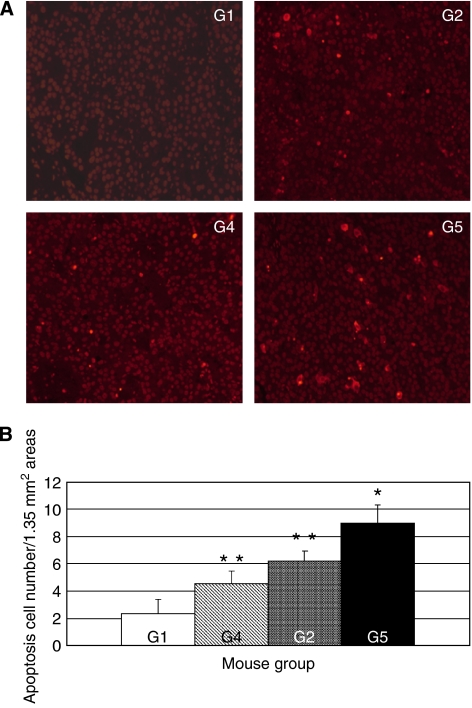
Incidence of apoptotic cells in TUNEL-stained 7-week tumour sections. The *In situ* Cell Death Detection Kit, TMR red was used to detect apoptotic cells in tumour sections. (**A**) Representative TUNEL-stained tumour sections from the G1 (control), G2 (5-FU), G4 (PEG-INF), and G5 (combination) groups. (× 100). (**B**) Average apoptotic cell numbers in TUNEL-stained tumour sections. Ten 1.35 mm^2^ areas in the HE-stained specimen that showed apoptotic cells in a relatively uniform density were selected for apoptotic cell counting. The 10 counts were then averaged to yield the data for the individual mice. The data shown are the average counts of the five mice in each group. The G5 tumours had significantly more apoptotic cells than all other groups, while the G2 and G4 tumours had significantly more apoptotic cells than the G1 tumours. The error bar represents s.d. ^*^Statistically significant difference compared with G1, G2, and G4 (*P*<0.05). ^**^Statistically significant difference compared with G1 (*P*<0.05).

Photomicrographs of the HE-stained tumour sections taken from the control and combination-treated animals at the end of the experiment did not reveal any marked differences between the two groups, although the control group tumours did show some necrosis, unlike the combination group tumours. This is because the control group tumours were significantly larger. We then examined the 7-week tumour sections for apoptosis by TUNEL analysis. Representative TUNEL-stained tumour sections are shown in Figure 2A. The tumours from the combination-treated animals had significantly more apoptotic cells (9.0±1.4/1.35 mm^2^ area) than the tumours from all the other groups (*P*<0.05, Figure 2B). The tumours from the animals treated only with 5-FU (6.2±0.7) or PEG-IFN (4.6±0.8) also had significantly more apoptotic cells than the control tumours (2.4±1.1) (*P*<0.05).

### Immunoprecipitation, immunoblot analysis and real-time RT–PCR

**Figure 3:**
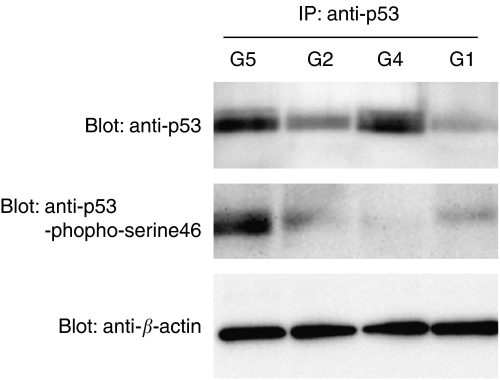
p53 levels and degree of p53 phosphorylation on serine46 in 7-week tumours. The extracted proteins (700 *μ*g) were subjected to immunoprecipitation with anti-p53 and immunoblot (Blot) analysis with anti-p53 or anti-p53-phospo-serine46 antibodies. As a control, the supernatants after p53 immunoprecipitation were directly subjected to immunoblotting using an anti-*β*-actin antibody. G1, control group; G2, 5-FU group; G4, PEG-IFN group; G5, combination group. The G4 and G5 tumours had higher p53 levels than the G1 and G2 tumours while the combination tumours showed more p53 phosphorylation on serine46 than the other tumours.

**Figure 4:**
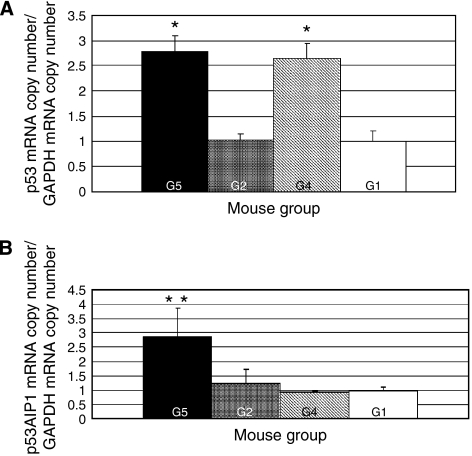
Quantitative RT–PCR analysis of p53 (**A**) and p53AIP1 (**B**) mRNA expression in 7-week tumours. Total RNA (200 *μ*g) isolated from the HepG2 tumours was subjected to TaqMan real-time PCR. The data were analysed on the basis of the *C*_t_ values of each sample and normalised relative to the GAPDH mRNA levels. The G4 and G5 groups showed increased p53 mRNA expression in their tumours compared to the G1 and G2 groups. The G5 tumours showed significantly increased *p53AIP1* mRNA levels when compared with the other tumours. The error bar represents s.d. ^*^Statistically significant difference compared with G1 and G2 (*P*<0.05). ^**^Statistically significant difference compared with G1, G2, and G4 (*P*<0.05).

We next determined the p53 protein and mRNA levels in the 7-week tumours by Western blot analysis and real-time RT–PCR, respectively (Figure 3 and 4). The degree of p53 phosphorylation on serine46 protein and the *p53AIP1* mRNA levels were also measured by Western blot analysis and real-time RT–PCR, respectively (Figure 3 and 4).

The extracted tumour proteins (700 *μ*g) were immunoprecipitated by using an anti-p53 antibody and then immunoblotted with anti-p53 or anti-p53-phospho-serine46 antibodies. The combination- and PEG-IFN-treated tumours had higher p53 levels than the control and 5-FU-treated tumours. The degree of p53 phosphorylation on serine46 was also significantly higher in the combination tumours than in the tumours of the other groups, which showed equivalent serine46 phosphorylation (Figure 3). When we examined the phosphorylation of p53 on serine15 and serine20, none of the groups showed any differences (data not shown).

Figure 4A shows the expression of *p53* mRNA normalised to that of GAPDH. The expression of *p53* mRNA in the combination- and PEG-IFN-treated tumours was significantly increased about 2.7-fold and about 2.6-fold in comparison to that in the control tumours, respectively. In addition, the level of *p53AIP1* mRNA in the combination-treated tumours was significantly increased about 2.8-fold when compared with that in the control tumours (Figure 4B).

## DISCUSSION

Interferon-*α* treatment on its own has been known to have an antitumour effect (Biron, 2001; Belardelli *et al*, 2002; Clemens, 2003; Hisaka *et al*, 2004). Indeed, a previous study has shown that IFN-*α* directly prevents and delays hepatocarcinogenesis by suppressing pre-neoplastic cell proliferation although this study also found that this effect may depend in part on the induction of p21 via a p53-independent pathway (Nakaji *et al*, 2004). In addition, long-term, low-dose, intermittent IFN-*α* therapy was shown to successfully delay the clinical recurrence of HCC after radical RFA therapy (Sakaguchi *et al*, 2005). These observations are consistent with those of the present experiment, which revealed that treatment with PEG-IFN on its own quite efficiently suppressed tumour growth.

Their combination of IFN-*α* and 5-FU has been shown previously to be highly effective clinically (Sakon *et al*, 2002; Patt *et al*, 2003; Obi *et al*, 2006). This is supported by our own observation that combination therapy with IFN-*α* and 5-FU induced significantly more apoptosis and tumour growth inhibition than monotherapy. This suggests that when IFN-*α* and 5-FU are combined, they act additively or synergistically to inhibit HCC. The mechanism behind this effect has been explored by several studies. One study reported that IFN-*α* activates thymidine phosphorylase (TP), which then metabolises 5-FU at higher rates, thereby increasing the intracellular levels of the active metabolite 5-fluoro-2′-deoxyuridine 5-monophosphate (FdUMP) (Schwartz *et al*, 1992). Interferon-*α* may also enhance the anti-HCC effect of capecitabine in nude mice by a similar mechanism (Xiao *et al*, 2004). Another study found that IFN-*α* inhibits thymidylate synthetases (TS), thereby enhancing 5-FU activity (Dou *et al*, 2005). In addition, the combination therapy has been shown to impair HCC growth by delaying the cell cycle and inducing apoptosis, since IFN-*α*/5-FU-treated human HCC cell lines show cell accumulation in the G0/G1 phase and increased expression of the cell cycle-related protein p27^Kip1^ (Eguchi *et al*, 2000). Thus, it is possible that IFN-*α* and 5-FU may together induce cell cycle arrest and apoptosis. Various mechanisms may be involved in this effect. First, the known proapoptotic effect of 5-FU that arises from the DNA damage it causes may be augmented in an additive manner by IFN-*α*, which may induce apoptosis by triggering the IFN-*α*/*β* receptor, which can then activate the signal transducer and activator of transcription (STAT)1 protein; STAT1 in turn regulates the expression of the Bcl-2 family of apoptosis-related proteins. Supporting the possibility that this mechanism is involved in the superior efficacy of the combination therapy, it has been noted that the growth-inhibitory effect of combination therapy is particularly marked in cell lines that express high levels of IFN-*α*/*β* receptors (Kondo *et al*, 2000, 2005; Ota *et al*, 2005). IFN-*α* and 5-FU may also act synergistically to induce HCC apoptosis due to the ability of both molecules to modulate the tumour necrosis factor-related apoptosis-induced ligand (TRAIL)/TRAIL receptor-mediated cytotoxic pathway. Here, IFN-*α* elevates TRAIL expression by activating antitumour effectors while 5-FU enhances TRAIL-receptor expression by HCC cells (Yamamoto *et al*, 2004).

Our study here also suggests strongly that IFN-*α*/5-FU combination treatment induces apoptosis and suppresses HCC proliferation by activating mechanisms that involve p53. P53 plays an essential role in the induction of cell cycle arrest and apoptosis. That p53 may be involved in the superior efficacy of the combination treatment has been suggested previously by a study showing that the antitumour effects of 5-FU and cisplatin correlate with p53 activity (Longley *et al*, 2003; Mujoo *et al*, 2003). Moreover, the IFN-stimulated gene factor3 (ISGF3) formed by IFN-*α* has been shown to induce *p53* gene expression by binding to at least two IFN-stimulated response element (ISRE) sites in the *p53* gene (Takaoka *et al*, 2003; Vilcek, 2003). We found that while the HepG2 tumours of PEG-IFN- and combination-treated animals showed significantly elevated p53 protein and mRNA levels, only the combination-treated tumours showed increased p53 phosphorylation at serine46 and elevated *p53AIP1* mRNA levels. P53AIP1 occurs only upon severe DNA damage, is activated by p53 after the latter is phosphorylated at serine 46, and appears to control the apoptosis-inducing function of p53 (Oda *et al*, 2000). These observations together suggest that upon combination therapy, PEG-IFN increases p53 protein levels while 5-FU induces tumour cell DNA damage that activates the p53 molecules generated by PEG-IFN. This may be responsible, at least in part, for the superior apoptosis-inducing and tumour-inhibiting effects of this treatment regimen.

Notably, in the present experiment, none of the treatments altered the degree of p53 protein phosphorylation at serine15 or serine20 (data not shown). After DNA damage, p53 phosphorylation first occurs at serine15 or 20, which stabilises and activates p53; p53 then binds to the promoters of G1 arrest-related genes such as p21^waf1^ and DNA repair-related genes such as p53R2 to induce their expression. If the DNA damage is severe enough, p53 then becomes phosphorylated at serine46 and induces apoptosis. Since only the combination group showed significantly increased serine46 phosphorylation, this suggests that the 5-FU component of this treatment regimen caused such severe DNA damage that the cells could not recover by inducing G1 arrest and DNA repair, thus resulting in serine46 phosphorylation and HCC apoptosis.

In this study, to determine the role of p53 plays in PEG-IFN/5-FU combination treatment efficacy, we selected the HepG2 cell line, as its p53 molecule is functional and does not bear mutations. Clearly, cell in which p53 is mutated and dysfunctional or even lacking may be significantly less susceptible to PEG-IFN/5-FU combination therapy than HepG2. Indeed, when Takaoka *et al* (2003) compared the effect of combination therapy on the HLE cell line, in which p53 is dysfunctional, and HepG2 *in vitro*, they found that the therapy killed HepG2 but not HLE. Thus, future studies should examine whether p53-mutated or -negative tumour cells can be susceptible to combination therapy *in vivo*.

In conclusion, recent studies have reported that IFN/5-FU combination therapy can effectively treat patients with advanced HCC (Sakon *et al*, 2002; Obi *et al*, 2006). Our present study reveals PEG-IFN/5-FU combination therapy operates at least in part by enhancing the proapoptotic function of p53.
